# Stroke Coordinators' Perspectives on Sustaining Use of Fever, Sugar (Hyperglycaemia) and Swallow (FeSS) Protocols. Process Evaluation Using a Sustainability Framework

**DOI:** 10.1111/jan.70125

**Published:** 2025-08-05

**Authors:** Kelly Coughlan, Oyebola Fasugba, Simeon Dale, Kelvin Hill, Dominique A. Cadilhac, Elizabeth McInnes, Sandy Middleton

**Affiliations:** ^1^ Nursing Research Institute ‐ St Vincent’s Health Network Sydney, New South Wales & St Vincent’s Hospital Melbourne, Victoria Australia; ^2^ School of Nursing, Midwifery and Paramedicine Australian Catholic University Sydney New South Wales Australia; ^3^ Stroke Foundation Sydney New South Wales Australia; ^4^ Stroke and Ageing Research, Department of Medicine School of Clinical Sciences at Monash Health, Monash University Clayton Victoria Australia

**Keywords:** fever, guidelines, hyperglycaemia, implementation, stroke, sustainability, swallow

## Abstract

**Aim:**

To: (i) examine Stroke Coordinators' perspectives of factors influencing sustained adherence to evidence‐based protocols to manage Fever, Sugar (hyperglycaemia) and Swallow (FeSS) and (ii) compare findings between hospitals with consistently high FeSS Protocol adherence versus those with consistently low or variable adherence.

**Design:**

Qualitative descriptive process evaluation using in‐depth, individual semi‐structured interviews.

**Methods:**

Hospitals that participated in ≥ 3 national stroke audit cycles were ranked by mean adherence to FeSS Protocols and stratified by consistently high, low and variable adherence. Three hospitals from each adherence strata were purposefully selected after further stratification by (i) previous participation in a FeSS Intervention study; and (ii) location (state, remoteness). Inductive thematic analysis was undertaken, with themes mapped to factors from the framework to compare findings by adherence level and contextualise the findings in relation to sustainability.

**Results:**

Analysis of 14 interviews identified two *themes* [and sub‐themes]: (1) *Stroke Coordinator as sustainability champions and boundary spanners* [maintenance of implementation strategies; fostering working relationships, communication and influence] mapped to Workforce factors, organisational and Innovation‐specific factors; and (2) *Hospital executive and middle management respect of stroke specialty* [designated area for stroke care; recognition of stroke specialist nursing skills; previous FeSS Intervention study participation] mapped to Workforce and Political factors. Key differences by adherence groupings related to the Stroke Coordinator model, workplace configuration, and the impact of interdepartmental relationships and competing organisational directives.

**Conclusion:**

The Stroke Coordinator role was pivotal for sustained use of evidence‐based FeSS Protocols for acute stroke care, driving multidisciplinary collaboration.

**Impact/Implications:**

Internationally, many patients do not receive evidence‐based acute stroke care. Despite the proven benefits of the FeSS Protocols, consistent implementation remains a challenge. This study recognises the critical importance of a dedicated Stroke Coordinator for all acute stroke hospitals. Their advocacy for the use of evidence‐based interventions is key to improving stroke outcomes.

**No Patient or Public Involvement:**

This study did not include patient or public involvement in its design, conduct, or reporting as it focused solely on the professional experiences of stroke care providers.

**Trial Registration:**

ACTRN 12623000445673. Registered 1 May 2023

**Abbreviations**
ASNEN: (Australian Stroke Nurses Education Network) Education
AuSCR: Australian Stroke Clinical Registry
FeSS: Fever, Sugar, Swallow
QASC: Quality in Acute Stroke Care
QASCIP: Quality in Acute Stroke Care Implementation Project
T^3^
: Triage, Treatment and Transfer

**Summary**

- Previously collected quantitative data (to assess sustainability outcomes at multiple time points over a 6‐year period) were complemented by qualitative insights from end‐users regarding the sustainment of implementation strategies for FeSS Protocols.
- Stroke Coordinators were identified as sustainability champions for the use of FeSS Protocols to maintain strategies and foster relationships across disciplines and departmental boundaries.
- Four factors from the Sustainability of Innovation Framework were identified for sustaining high adherence to FeSS Protocols: workforce (having more than one stroke champion), organisational (effective working relationships, designated stroke speciality area and designated nursing staff), innovation‐specific (local adaptations) and political (organisational agreement before implementation).

## 1 Introduction

Evidence‐based recommendations for stroke care in hospitals are available to clinicians through practice guidelines (Stroke Foundation, n.d.). In 2017, a ‘Strong Recommendation’ to support the use of standardised protocols to manage Fever, Sugar (hyperglycaemia) and Swallow (FeSS) difficulties in patients experiencing an acute stroke was included in the Australian Clinical Guidelines for Stroke Management (National Stroke Foundation 2017). Recommendations for the management of these common post‐stroke complications are also included in international clinical practice guidelines (Powers et al. 2019; Royal College of Physicians 2016). Despite the existence of this clinical practice guideline, a recent national audit of in‐hospital stroke care reported a persistent evidence‐practice gap. The recommended prompt treatment of fever and hyperglycaemia was reported to be lower than the previously conducted audit cycle (2023:44% vs. 2021:50%; 2023:27% vs. 2021:29%), and 40% of patients were inappropriately given oral intake before a swallow screen or assessment had been performed (Stroke Foundation 2023). Similar evidence‐practice gaps in the management of these common acute stroke complications have also been reported internationally (Cassier‐Woidasky et al. 2024; Middleton et al. 2022). The significant variability in adherence across hospitals highlights inconsistencies in the use of FeSS protocols, the underlying causes of which are poorly understood.

## 2 Background

The Quality in Acute Stroke Care (QASC) Trial demonstrated a 15.7% reduction in death and disability following facilitated implementation of the FeSS Protocols (Middleton et al. 2011), instigating the recommendation in national clinical practice guidelines. Subsequent state‐wide scale‐up of the FeSS Protocols into stroke units and stroke services throughout New South Wales, Australia, reported significant improvements in protocol uptake (The QASC Implementation study [QASCIP], 2013–2014) (Middleton et al. 2016). Significant improvements were also demonstrated in the international upscale QASC Europe Study (2017–2021) in 64 hospitals within 17 countries (Middleton et al. 2022). However, upscale into non‐stroke‐specific settings in the Triage, Treatment and Transfer (T^3^ Trial, 2013–2016) was not significant in the context of competing demands of busy emergency departments (Middleton et al. 2019). The FeSS Intervention studies (QASC, QASCIP and T^3^) used active evidence‐based, multi‐faceted implementation strategies (clinical champions, barrier and enabler assessments, educational workshops and reminders) (Middleton et al. 2011, 2016, 2019). Beyond these studies, there have been no nationwide active implementation efforts to introduce the nurse‐led FeSS Protocols.

Passive implementation strategies (Vedel et al. 2018) to scale‐up this complex health innovation consist of inclusion in national clinical practice guidelines and self‐monitoring by voluntary participation in the biennial National Acute Stroke Audit and feedback cycles (hereinafter referred to as the Audit) (National Stroke Foundation 2017; Stroke Foundation 2020). FeSS variables have been included in the audit from 2013, permitting standardised monitoring in Australian hospitals. This quality improvement initiative is designed to report on adherence to recommendations outlined for in‐hospital acute care in National Clinical Guidelines for Stroke Management. Feedback is provided to hospitals as site‐specific reports with the ability to benchmark their performance against similar services across Australia (Stroke Foundation 2020).

Despite these active and passive efforts over the last decade to scale‐up use of FeSS Protocols, consistent adherence remains suboptimal. Our recent study of FeSS Protocol adherence evaluated national audit data (2015–2021) after the strong recommendation for the use of these protocols was included in the National Clinical Guidelines for Stroke Management (Coughlan et al. 2024). Although there had been some improvement, less than half of Australian patients were being cared for in accordance with these protocols. Additionally, a hospital's prior participation in the treatment arm of a FeSS Intervention study, and/or stroke‐unit care were independently associated with greater adherence to FeSS Protocols (Coughlan et al. 2024).

It remains unclear why some hospitals consistently perform well in providing care in accordance with FeSS protocols while others do not. Sustainability has been identified as a research priority in implementation science (Proctor et al. 2015) with growing recognition that strategies required for sustainability may differ from those used for initial implementation (Bagot et al. 2020, 2023). Sustainability is defined as a process that occurs after a specific period of time whereby a program, clinical intervention, and/or implementation strategies continue to be delivered and/or individual behaviour change is maintained. The program and individual behaviour change may evolve or adapt while continuing to produce benefits for individuals/systems (Moore et al. 2017). Our previously published study has examined sustainability (the use of FeSS Protocols [clinical intervention] in Australian hospitals at defined points in time), and this study, undertaken as a process evaluation, is designed to examine sustainment (continued use of implementation strategies) (Wiltsey Stirman et al. 2012).

### 2.1 Aims

We aimed to: (i) examine Stroke Coordinators' perspectives of organisational and individual factors influencing sustained adherence to FeSS Protocols in hospitals that participated in the 2015–2021 audit cycles; and (ii) compare findings between hospitals with consistently high FeSS Protocol adherence versus those with consistently low or variable adherence.

## 3 Methods

### 3.1 Study Design

This study adopted a qualitative descriptive process evaluation underpinned by a critical realist paradigm. Critical realism acknowledges that while an objective reality exists, our understanding of it is shaped by social, cultural, and contextual influences (Clark et al. 2008). Process evaluations aim to understand the intervention process and factors associated with effective knowledge translation in a particular context (Rantsi et al. 2022). Previous process evaluations have accompanied FeSS Intervention studies (McInnes et al. 2024, 2020), however, the factors influencing strategies for sustaining the use of FeSS Protocols outside of study conditions are yet to be explored. Qualitative methods are the most common methods for process evaluation studies (Rathod et al. 2024). Qualitative descriptive research aims to discover and understand a phenomenon, process, or the perspectives of those involved. The focus is on describing rather than explaining, making it especially suitable for researching poorly understood areas (Sandelowski 2000; Bradshaw et al. 2017). A qualitative descriptive process evaluation in Australian hospitals that participated in the 2015–2021 Audits using in‐depth, individual semi‐structured interviews with Stroke Coordinators was conducted to explore factors that influence FeSS Protocol adherence. The reporting of this study was guided by the COREQ (COnsolidated criteria for REporting Qualitative research) guidelines (Tong et al. 2007).

### 3.2 Setting

#### 3.2.1 Eligibility

Australian hospitals that participated in three or more audit cycles in 2015, 2017, 2019 or 2021. In general, hospitals that had 50 or more stroke admissions each year were invited to participate in each audit.

#### 3.2.2 Sampling Method

Hospitals were ranked based on their mean FeSS adherence composite score for each audit cycle. The composite score is the proportion of patients who received all six treatment elements of the FeSS Protocols among the eligible patients for these processes of care: (i) prompt treatment with paracetamol for fever; (ii) prompt treatment with insulin for hyperglycaemia; (iii) swallow screen or assessment within 24 h; (iv) swallow screen or assessment before receiving oral food or fluids; (v) swallow screen or assessment before receiving medication; and (vi) swallow assessment if they failed a swallow screen (Coughlan et al. 2024).

Hospitals were stratified into consistently high adherence (≥ 25% above the overall mean FeSS composite score for three or more audit cycles); consistently low adherence (≥ 25% below the overall mean FeSS composite score for three or more audit cycles) and variable adherence (one or more audit cycles in the low adherence threshold and one or more audit cycles in the high adherence threshold).

The hospitals were then stratified by (i) previous participation in any of the FeSS Intervention studies (Middleton et al. 2011, 2016, 2019), and (ii) according to region (state) and remoteness (Area based on the Accessibility and Remoteness Index of Australia [ARIA+]; Australian Bureau of Statistics 2021). The use of remoteness was a proxy for stroke units, as access to these is markedly lower in regional and remote areas compared to major city hospitals (Stroke Foundation 2023). Additionally, the QASC and QASCIP FeSS Intervention studies were conducted in one Australian state only. Purposeful selection from various Australian states and territories, and the additional strata were to ensure representation of a broad range of hospitals to comprehensively explore any differences in factors identified for sustainability.

Three hospitals were purposively selected from each of the FeSS Protocol adherence groups. This strategy of ‘extreme sampling’ is an efficient way to evaluate information‐rich exemplars of extremes of the variation in the target population (Patton 2002). Hence, a hospital was ranked as ‘consistently’ high or low FeSS Protocol adherence using data from three or more of the four audit cycles. Extreme sampling has also been used in a previous FeSS Intervention process evaluation study, but not over this many time points (McInnes et al. 2020). Those with variable adherence were included to provide insight into potential reasons for variation. Sample size was guided by the concept of information power rather than data saturation. The premise of this method is that the more relevant information the sample holds, specific to the study aims, the lower the number of participants are needed (Malterud et al. 2015). Purposive extreme sampling, supported by established theory from theoretical frameworks (Fox et al. 2015; Greenhalgh et al. 2004) was expected to provide sufficient information regarding recruitment numbers and dialogue quality (Malterud et al. 2015).

#### 3.2.3 Clinician Eligibility

The Stroke Coordinator role is recognised globally as a specialist position (usually nursing), with several years' experience of directing or providing acute care for stroke patients (Purvis et al. 2021; Aroor et al. 2024). There is a wide range of alternative titles for this role (e.g., Clinical Nurse Consultant, Acute Stroke Nurse, Stroke Nurse Navigator) herein collectively referred to as Stroke Coordinators. Common responsibilities of this role consist of educating interdisciplinary staff and patients, demonstrating clinical leadership, and contributing to the development and implementation of protocols that promote standardised, evidence‐based care (Purvis et al. 2018). As FeSS Protocols are designed to be nurse‐initiated, these senior nursing clinicians were considered the most likely personnel to be directly involved in implementing new guidelines, and sufficiently knowledgeable regarding factors that may influence the sustainment of these strategies after initial implementation.

In hospitals that did not have a Stroke Coordinator, participation from the Nurse Unit Manager of the stroke unit or stroke service (or equivalent stroke clinician leader) was sought. We aimed to interview a minimum of one person per hospital, but also asked participants during the interviews if they were able to identify one other stroke clinician (from any discipline) who was involved with the implementation of FeSS Protocols within their hospital that could offer additional insights.

#### 3.2.4 Clinician Exclusion Criteria

New employees of the hospital (less than 6 months) were excluded. In hospitals without a Stroke Coordinator, Nurse Unit Manager (or equivalent stroke clinician leader) the hospital was excluded.

### 3.3 Recruitment

The publicly available audit report includes the name of a site contact for each participating hospital. This person was contacted to obtain the name of the Stroke Coordinator (or equivalent stroke clinician leader) where this role was not filled by the named contact. The Stroke Coordinator (or equivalent stroke clinician leader) for each of the nine hospitals was invited via email to participate in a semi‐structured video‐conference interview. If there was no response to this invitation, a follow‐up email was sent 2 weeks later, with a telephone follow‐up a week after this. If participants were able to identify one other stroke clinician (from any discipline) who was involved with the implementation of FeSS Protocols within their hospital, they provided the clinician's contact details.

### 3.4 Theoretical Framework and Interview Schedule

The Sustainability of Innovation Framework (Fox et al. 2015), is one of the eight recommended models, theories or frameworks to evaluate and interpret concepts of sustainability of evidence‐based practices within acute care contexts (Nadalin Penno et al. 2019). Grounded in the conceptual Model for Considering the Determinants of Diffusion, Dissemination, and Implementation of Innovations in Health Service Delivery (Greenhalgh et al. 2004), and the Dynamic Sustainability Framework (Chambers et al. 2013), this theoretical framework was deemed the most relevant for focused analysis on sustained adherence. Semi‐structured interview questions and prompts were guided by this framework and one of the more detailed models it was founded on (Supporting Information S1) (Greenhalgh et al. 2004). Individual factors and organisational factors influencing clinician adherence to FeSS Protocols were explored, and details on intervention strategies used to implement the protocols were also collected. The interview schedule differed slightly for those hospitals that reported that they had not actively used any implementation strategies, as participants would then only be able to hypothesise on potential factors that influenced the decision not to implement (Supporting Information S1).

### 3.5 Data Collection

Interviews were conducted and recorded via video conference (Microsoft Teams, v25072.1611.3570.1995). Participants were not informed of their hospital's FeSS Protocol adherence grouping; however, they were aware of the purpose of the study.

### 3.6 Data Analysis

Transcripts were deidentified and imported into NVivo software (version 12). Participant demographics are presented using descriptive statistics. Analysis was conducted using inductive thematic analysis (Castleberry and Nolen 2018) with the theoretical frameworks used to guide the interview schedule. A formal coding template was not developed a priori as data were analysed inductively, which involved iterative coding phases and theme refinement processes (Castleberry and Nolen 2018; Creswell and Báez 2020; Elliott 2018). The coding process was conducted by one researcher with regular meetings with expert qualitative researchers within the team to reach consensus on emergent themes. Themes were mapped to factors from the Sustainability of Innovation Framework (Fox et al. 2015) to support deeper interpretation and contextual relevance. The factors in this framework were derived from two other well‐established conceptual frameworks (Greenhalgh et al. 2004; Chambers et al. 2013) which were also consulted for further clarification as required. Comparative data analysis between hospitals based on their adherence was considered against the factors from the conceptual framework and in alignment with the research questions. Results are presented as themes constructed from sub‐themes and how these relate to factors identified in the literature to support sustainability from the Framework.

### 3.7 Ethical Considerations

Ethics approval for the study was obtained from St Vincent's Hospital Human Research Ethics Committee, Sydney (2023/ETH00446) on the 20th April 2023. Participants provided written informed consent.

### 3.8 Rigour and Reflexivity

Strategies were implemented to enhance trustworthiness, consistency, and applicability. Researcher bias was addressed by interviewer self‐disclosure and regular team discussions during analysis. Transcripts were verified against video recordings to ensure accuracy and deepen data familiarity. Direct participant quotes supported representativeness, while systematic documentation of coding and theme development maintained analytical consistency. Qualitative expertise was available within the team, and contextual details were included to support the transferability of findings to similar clinical settings (Cypress 2017; Noble and Smith 2015).

## 4 Results

Of the 131 hospitals that participated across one or more of the audit cycles, 109 had participated in three or more of these cycles. Fifty‐two hospitals had participated in a FeSS Intervention study (QASC 2005–2010, QASCIP 2013–2014, T^3^ 2013–2016). Six hospitals were ranked with consistently high adherence and eight were ranked with consistently low adherence. Fourteen hospitals had variable adherence across the audit cycles. All six hospitals ranked with consistently high adherence had stroke units and five had previously participated in a FeSS Intervention study. Of the eight hospitals that were ranked with consistently low adherence, only two had a stroke unit and one had previously participated in a FeSS Intervention study. Five of the 14 hospitals with variable adherence had previously participated in a FeSS Intervention study and 12 had a stroke unit.

**FIGURE 1:**
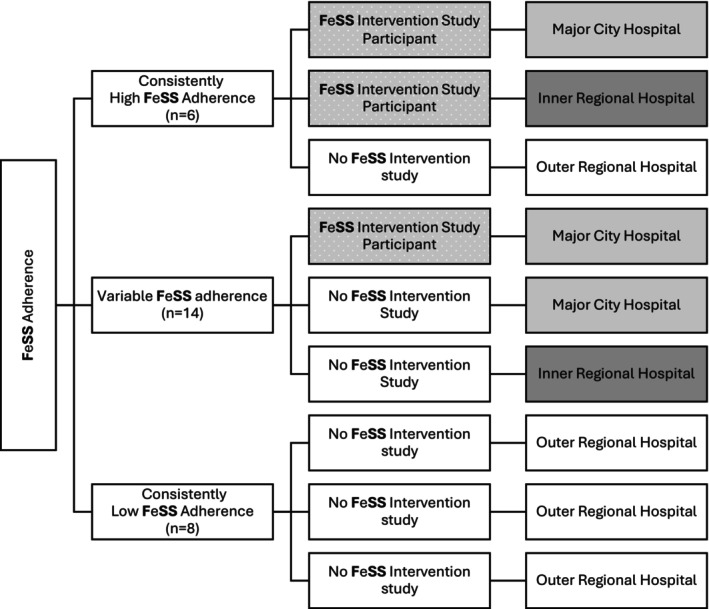
Study sample by FeSS Protocol adherence groups, previous FeSS Intervention study participation and location (remoteness) (Australian Bureau of Statistics 2021).

Three hospitals from each of the consistently high, consistently low adherence, and variable FeSS Protocol adherence were purposively selected after further stratification according to prior participation in a FeSS Intervention study and from geographically diverse areas where results in these groupings allowed (e.g., only one of eight hospitals in the consistently low adherence group had previously participated in a FeSS Intervention study but no longer had a stroke unit or Stroke Coordinator) (Figure 1). Given the risk of identification of hospitals in smaller States or Territories, the location is not presented; however, interviews were conducted with Stroke Coordinators from all States and Territories except for one.

### 4.1 Participant Characteristics

Six of the nine Stroke Coordinators identified an additional participant from their hospital. Of a possible 15 interviews, 14 were completed between September 2023 and January 2024 (one interview participant failed to respond after several follow‐up attempts). The interviews ranged from 15 to 49 min in length, with a mean duration of 31 min. All were registered nurses, excluding one occupational therapist and one stroke neurologist, and 79% (*n* = 11) of interview participants were female (Table 1). The median years of clinical experience were 21 (range 9–35) and the median years in their current role were 7.5 (range 1.5–17).

**TABLE 1:** Participant characteristics.

Health profession	Gender	Years of clinical experience	Years in current role
Nursing	Female	31	17
Nursing*	Female	16	16
Nursing	Male	34	3
Nursing*	Female	35	7
Nursing	Female	24	8
Occupational Therapist	Female	10+	5
Nursing	Female	13	1.5
Nursing*	Female	20+	8
Nursing	Female	9	3
Stroke Neurologist*	Male	25	7
Nursing	Male	21	7
Nursing	Female	30	11
Nursing	Female	14	10
Nursing*	Female	16	16

* Secondary interview participant.

### 4.2 Major Themes Mapped to Sustainability of Innovation Framework

Two themes were constructed from several sub‐themes and mapped to factors from the framework (Table 2). Participant quotes are used to illustrate the findings.

**TABLE 2:** Alignment of themes and sub‐themes with factors from the Sustainability of Innovation Framework.

Themes and sub‐themes	Sustainability of Innovation Framework factors
*Stroke Coordinator as sustainability champions and boundary spanners* Maintenance of implementation strategies Fostering working relationships Communication and influence	*Workforce*: (i) Employment models (i.e., a single staff member Stroke Coordinator model compared to a team of clinical champions) can influence the sustainment of implementation strategies. (ii) Working relationships require effective interpersonal skills by the Stroke Coordinator to act as a boundary spanner between departments/disciplines/organisations to foster network collaboration. *Organisational*: (i) Implementation strategies were initiated and leveraged from existing systems. (ii) Sustainment of implementation strategies requires ongoing maintenance to embed FeSS Protocols into clinical practice. (iii) Operational governance requires effective communication and maintenance of relationships with key stakeholders that share an understanding and value the use of FeSS Protocols. *Innovation‐specific*: (i) Site‐specific adaptations of FeSS Protocols after initial implementation for optimisation in response to context, enable sustainability.
*Hospital executive and middle management respect of stroke specialty* Designated area for stroke care Recognition of stroke specialist nursing skills Previous FeSS Intervention study participation	*Workforce*: Hospital executive and middle management support and recognition of stroke expertise are required. This necessitates: (i) a designated area (e.g., stroke unit/service) where the FeSS Protocols align with the values and needs of the multidisciplinary team; and (ii) recognition that personal motivation, capacity, and competencies are different for stroke nursing compared to other speciality areas and levels of patient acuity. *Political*: (i) a focused organisational approach before implementation of FeSS Protocols (e.g., agreement to participate in a FeSS Intervention study) enhances sustainability. (ii) It is important to be aware of other competing organisational directives before trying to implement an evidence‐based intervention.

#### 4.2.1 Stroke Coordinator as Sustainability Champions and Boundary Spanners

The Stroke Coordinator role was identified as the lead for initial implementation of FeSS Protocols and for maintenance and sustainment of these strategies used in their individual stroke services. As senior stroke nurse leaders, they assumed responsibility for driving evidence‐based practice and acted as a ‘boundary spanner’ to communicate across disciplines and departmental boundaries. This required the use of interpersonal skills to foster working relationships and coordinate organisational‐level approaches to acute stroke care.

##### 4.2.1.1 Sub‐Themes

###### 4.2.1.1.1 Maintenance of Implementation Strategies

Education and training were common strategies employed for initial and ongoing implementation of FeSS Protocols into local stroke service processes and everyday clinical practice. A standardised document or care plan with organisational approval for use, specific to individual hospitals, was the predominant method to support these approaches. The names for these varied widely (policy, stroke care plan, pathway, checklist, protocol). Generally, this document was developed in line with the national clinical guidelines for acute stroke management that became a reference for use within the stroke service when requesting medical review and/or intervention:

*We have pretty good adherence to our swallowing, the fever and sugar only started probably with the FeSS protocols. We incorporated into our initial management in ED and then and into our stroke pathway in the ward*. *[High Adherence 1]*

*We have included the guidelines for FeSS Protocols into our governance for stroke unit care which can be easily accessed on the intranet*. *[Variable Adherence 3]*

Participants from hospitals with high FeSS Protocol adherence reported that these documents were used by nursing and medical staff to support care in accordance with FeSS recommendations:

*It's on our intranet, if I put in stroke patient then all of the procedures, care paths, come up for you and you can pick it out. Every three years they get reviewed. FeSS has been added to the medical template in EMR*. *[High Adherence 2]*

*The junior doctors have a broad range of things they have to know in multiple patients … If we've got a stroke pathway and a framework, you can kind of say, well, here it is. And this is why we do it … they're very open to it*. *[High Adherence 1]*

The existence of a multidisciplinary shared care pathway or document did not necessarily facilitate adherence, as the Stroke Coordinator from a hospital with consistently low adherence also reported the use of a state‐wide stroke protocol designed for multidisciplinary and multiple departments to use:

*It follows the stroke key guidelines … takes the doctors and the nursing staff through everything they have to do in that ED setting and then onto the acute ward and discharge planning* *[Low Adherence 1]*

Leverage from an existing process to include FeSS Protocols into clinical practice was perceived positively in relation to the transparency and consistency of requirements for stroke care in all hospitals in the same region. However, upscaling of a local stroke protocol on a larger scale was perceived negatively by the Stroke Coordinator from a hospital with consistently low adherence due to the significant time delays associated with the increased levels of consultation required for any updates. In some circumstances, this meant the protocol was then unavailable during the consultation and review period:

*Having a district wide pathway meant we had to go through all that consultation. How can we actually just evolve what we've got instead of taking it away and saying no, you can't use that anymore, and then we've got nothing for this period of time* *[Low Adherence 2]*

Similarly, frustration with broader organisational processes was described by the Stroke Coordinator from a low‐adherence hospital in relation to their requirement to follow a generic protocol for all acute inpatient admissions in that region, rather than a stroke‐specific protocol. There had been some recent success in negotiating approval for the use of a stroke thrombolysis and retrieval pathway, but a protocol specific to acute stroke management (for patients not eligible for stroke thrombolysis) was yet to be approved:

*It is only thrombolysis and early assessment, and the other part of the protocol is for all general medicine patients, but not specific for stroke*. *[Low Adherence 3]*

The need to de‐implement outdated recommendations and update stroke pathways/protocols to manage service expansion (such as stroke telemedicine) or to maintain alignment with current national stroke clinical practice guidelines generated much discussion. For smaller stroke services, the evolution of the stroke telemedicine model was perceived positively, as protocols for this service were developed collaboratively with the larger and better‐resourced stroke hospitals. The Stroke Coordinator from a hospital with variable adherence described how the role itself evolved with this service:

*The Stroke Coordinator role is basically a new role for my organisation that has just came up when the Telestroke service has been implemented. We only started thrombolysis as part of the telestroke service implementation last year. Now with the new stroke pathway, they're just learning or starting to become aware of the FeSS protocol …* *[Variable Adherence 3]*

The incorporation and maintenance of these documents in electronic medical records (EMR) had varied perceptions. Being able to add FeSS Protocols to medical admission documentation templates was perceived positively (as it facilitated a prompt for medical staff to prescribe care in relation to these protocols). However, the additional governance and approval processes in relation to this technology integration were perceived negatively as it was thought to contribute to significant time delays:

*We have a stroke patient acute procedure for non‐thrombolysed patients on our intranet. There is an electronic care plan developed for this, but we've never implemented because of the EMR delays. We keep hearing its coming, but it's never really happened*. *[High Adherence 2]*

*Initially, the plan was to put it up in the EMR but this will take a very long time. As far as I'm aware, a lot of the stroke pathway forms are in paper form because it's easier to change it because of the quick change in Stroke Foundation guidelines*. *[Low Adherence 2]*

The Stroke Coordinator from a hospital with variable adherence described an update to their stroke pathway design that may have had a positive influence on their improved adherence. The revised design was aligned with national stroke clinical practice guidelines and now required documentation of compliance with this pathway for each nursing shift.

*FeSS was all sort of built into our pathway beforehand. Now there has to be some accountability that these things were done, for each shift*. *[Variable Adherence 1]*

The existence of a stroke unit to centralise acute stroke care was perceived to facilitate a cohesive approach to patient care, confirming alignment with shared goals of the organisation for evidence‐based acute stroke care. The hospital‐approved policies and protocols (adapted to include FeSS Protocols) were reported to be uniformly applied and regularly reviewed for currency with the latest evidence. The relocation of a stroke unit was described as destabilising and how the absence of a stand‐alone stroke unit impacted the Stroke Coordinators ability to reinforce and monitor compliance with stroke protocols:

*We've joined the whole Neurosciences ward as one and when that happened, we've had a fairly large turnover of staff. The stroke unit looked feral and old, but the systems, even though I thought they didn't work very well back then, I think they probably did because we were all in one area together*. *[High Adherence 3]*

*We don't have a stroke unit; protocols are not updated (the stroke pathway has not been updated since 2016). If they're not transferred out to a stroke unit, they can go basically anywhere …* *[Low Adherence 2]*

###### 4.2.1.1.2 Fostering Working Relationships

The ability of the Stroke Coordinator to effectively maintain working relationships across departmental boundaries and disciplines was reported as being essential for sustained adherence to FeSS Protocols. Predominantly, these were specific to the emergency department, speech pathology, and medical colleagues:

*I would have to say I didn't get a lot of buy in or support from ED in regard to FeSS or time to imaging or any of those sorts of markers. And that's what's been a real challenge over the last couple of years … I just got told by ED: I'm sorry it's not a priority*. *[Low Adherence 1]*

*So the swallow, it has been a problem because there is not always someone available in the stroke unit and in ED or in other medical wards to perform dysphagia screening. They are not trained to do it, and they have been always reluctant to be trained in swallow screening* *[Low Adherence 3]*

The relationship with the speech pathology department was identified as a positive influential factor for sustained adherence to FeSS Protocols, especially where there were shared arrangements for education and training, data collection and feedback. Another key relationship highlighted was with medical leadership and inconsistent support in relation to sustained adherence to FeSS Protocols. Frequently, the Stroke Coordinator assumed some responsibility for orienting new and junior medical staff. However, if acute stroke management in accordance with FeSS Protocols was not reinforced by the medical leadership group, then sustainment of implementation strategies was difficult:

*I could kind of remind the doctors (we have a new one every six weeks). But then after hours, obviously there was no one there to prompt people. Our consultants obviously don't feel strongly about it* *[High Adherence 3]*

*My biggest issue would be the doctors not being aware, even though I give them orientation, the protocols and the consultants will say they're not aware. The doctors don't enforce our words*. *[Variable Adherence 2]*

The Neurology Advanced Trainee (3‐year speciality fellowship post‐basic physician training) was an exception to this finding, and was reported as someone who valued the evidence for the use of FeSS Protocols and supported their use:

*The neuro advanced trainees are very good, because they've come from other areas as well. So they'll give us some feedback too. If you say, what's the reasoning behind this? And they'll fill you in, on the latest research*. *[High Adherence 1]*

*Our advanced trainee at the moment is from XXXX, so they document, you know, as per FeSS protocols*. *[High Adherence 2]*

###### 4.2.1.1.3 Communication and Influence

Data collection is a common task for most Stroke Coordinator roles (Purvis et al. 2021) and widely reported in this study to be onerous. Those Stroke Coordinators that collected data for the audit cycles reported to have also been responsible for communicating those results back to the multidisciplinary team. The Stroke Coordinator (from a hospital with consistently low FeSS Protocol adherence) who did not perform data collection for the audit cycles was unaware of their hospital's participation, as the results had never been shared with the nursing staff:

*So, we've never done that [the acute Audit] on the ward, just the rehab Audit normally, I'm not sure who would collect that data because I've never done any*. *[Low Adherence 3]*

Participants were asked about any additional data collection (FeSS variables) outside of the audit cycles, and if/how these results were communicated to the nursing staff and broader multidisciplinary team. Additional data on FeSS variables was contributed to local databases and/or the Australian Stroke Clinical Registry (AuSCR), which has an optional dataset (as of July 2019) to collect continuous FeSS variables prospectively:

*We know where our gaps are because we do look at our AuSCR data a lot and that's why we signed on for the FeSS (optional dataset in AuSCR)*. *[Variable Adherence 3]* .

*I do monthly reports about the code stroke process to the medicine department and six monthly reports on adherence to the stroke protocol*. *[Variable Adherence 2]*

Strong links to professional networks were reported by Stroke Coordinators from hospitals with high and variable adherence as a factor perceived to enhance confidence in advocating for adherence to FeSS Protocols in their stroke services. Some of these networks were region‐specific, and membership in a national stroke nursing network (the Australian Stroke Nurses Education Network [ASNEN]) was also common. These forums were described as an opportunity to discuss common issues in relation to FeSS Protocol implementation and sustainability:

*We've talked about FeSS quite a lot and it's been something that we've tried to make a bit of a focus through some of the education that we've done as well. But certainly, I think the barriers are fairly similar across the board*. *[Variable Adherence 1]*

*You kind of need to go to these things (ASNEN stroke nurse leaders' day) to learn that you're not just an utter failure … you know, it's big to implement three protocols at one time*. *[High Adherence 3]*

##### 4.2.1.2 Mapping to Factors From the Sustainability of Innovation Framework

The first theme of the Stroke Coordinator as sustainability champions and ‘boundary spanners’ was constructed from sub‐themes of maintenance of implementation strategies, fostering working relationships and communication, and influence. Leverage and adaptation of hospital‐specific existing systems and tools by Stroke Coordinators to embed routine use of FeSS Protocols into clinical practice required ongoing maintenance for their sustained use. Underlying many of the reported issues were *Organisational Factors*. Obtaining operational governance for these strategies was not a one‐off process. As technology and the services offered by the hospital evolve, implementation strategies have to be flexible enough to accommodate these changes. Maintenance of interdepartmental relationships was identified by the Stroke Coordinators from hospitals with high adherence as essential to ensure the strategies in place for FeSS Protocols were not overlooked during any changes or upgrades within their service. Furthermore, those who were able to navigate the complexities of new technological innovations, such as telestroke and EMR, effectively leveraged these tools and new ways of working to incorporate FeSS Protocols to facilitate sustained adherence.

Although the protocols are designed to be nurse‐led, they require endorsement from medical and speech therapy colleagues. Consideration of the Stroke Coordinator's interpersonal influence and ability to foster network collaboration with relevant stakeholders as the sustainability champion highlights issues in relation to *Workforce Factors*. At the organisational level, the risk of a single Stroke Coordinator service model makes any implementation efforts vulnerable if this staff member is unavailable. In the absence of peers to share responsibilities of overcoming barriers to sustainment of implementation strategies, this critical staffing resource and strategies are compromised:

*We don't have anybody in the district who's got stroke experience, or a stroke champion or Clinical Nurse Specialist. So that lack of nursing expertise in stroke nursing is very difficult as well*. *[Low Adherence 2]*

*I don't have anybody else; our Clinical Nurse Educators are largely not stroke, I bear the main load … After hours, obviously there is no one there to prompt people. We had the protocols but obviously unless someone is there to push it, it didn't really happen*. *[High Adherence 3]*

When considering this workforce factor for hospitals with variable adherence, an improvement in adherence may have coincided with the expansion of the single Stroke Coordinator role to a 24‐h model of care. Mitigating some of the risk associated with reliance on a single staff member Stroke Coordinator service model was also addressed by identifying stroke champions in other departments. These clinicians self‐identified as having an interest in acute stroke care and were willing to learn about managing FeSS complications. This shared understanding facilitated their championing of the use of FeSS Protocols in their respective departments:

*I've created some ED and ICU stroke champions in those key stakeholder areas, some of them have fully completed the ASSIST (dysphagia screening tool) training and others are now doing it*. *[Variable Adherence 3]*

*I think with the turnover of staff, get your champions actually working actively to help you so that it's not all dependent on the one senior nursing stroke lead*. *[Variable Adherence 1]*

*Innovation‐specific* factors for sustainability relate to the acceptability, quality and safety of the innovation to stakeholders. There were some reports of a lack of medical leadership to enforce the FeSS Protocols, particularly in relation to the use of insulin for the treatment of hyperglycaemia. In response to the perceived risk or uncertainty, several adaptations to the FeSS Protocols to suit local context were in place (e.g., use of subcutaneous insulin instead of intravenous infusions and/or review by the endocrinology team).

#### 4.2.2 Hospital Executive and Middle Management Respect of Stroke Specialty

The second theme was the essential principle of hospital executive and middle management support and respect for stroke as a distinct speciality area. The need for a designated area for stroke care and recognition of stroke specialist nursing skills was a sub‐theme fundamental to respect for the knowledge required to effectively manage and treat acute stroke patients. Previous FeSS Intervention study participation was another sub‐theme pertinent to this topic as the benefits of a focused organisational agreement before attempts to implement FeSS Protocols became evident.

##### 4.2.2.1 Sub‐Themes

###### 4.2.2.1.1 Designated Area for Stroke Care

Workplace configuration and the presence of a dedicated stroke unit featured prominently as a topic during interviews. Of the hospitals with consistently low FeSS Protocol adherence, only one was equipped with a stroke unit. The Stroke Coordinators from hospitals with high and variable adherence came from stroke units that operated independently as autonomous specialist departments:

*We've only got 5 beds, and we're completely separate to the rest of the ward, so the nursing staff only work in the stroke unit. I think that the rest of the hospital has that respect for our unit. And they're very good at allowing us to manage the unit ourselves* *[High Adherence 1]*

However, constant negotiations are required to reserve these beds for patients with stroke and to have stroke specialist staff assigned to their care was emphasised:

*Ringfencing of the beds is a daily challenge, to try and keep patients ‘cohorted’ together, I've been trying to have the roster ringfenced as well, so you have specific nurses in that acute space* *[High Adherence 2]*

The Stroke Coordinator from the only hospital with low FeSS Protocol adherence that had a stroke unit described the issues with inconsistent personnel for staffing (attributed to the hospital's location and transient population). Additionally, the patients in this stroke unit were not always managed by the neurology team unless they were the admitting team:

*The problem is that the patients are admitted under General Medicine so that is a different team of doctors every day that admit patients with stroke. This creates a bit of confusion, even if we lead the decisions, they are not immediately our responsibility*. *[Low Adherence 3]*

###### 4.2.2.1.2 Recognition of Stroke Specialist Nursing Skills

Acknowledgement and recognition of the skills required to work in specialist stroke units varied between hospitals. Demonstration of competency was required by units with high and variable adherence compared to the one stroke unit with low adherence, which was based on length of employment only:

*You can't work in our stroke bay, until you've done the transition program for stroke neurology and the telemetry package. No graduate nurses [work in this area] until it is their second year*. *[High Adherence 2]*

*Everyone who worked in the stroke unit had to have certain modules ticked off. They were given an orientation book that was very heavily FeSS influenced*. *[Variable Adherence 1]*

*New staff are not allowed into the stroke unit until they've been on the ward for over a year*. *[Low Adherence 3]*

There was significant discussion in relation to the stroke unit and other clinical specialities that shared the same workspace. Co‐locating the acute stroke patients with sub‐acute inpatient rehabilitation patients was described as a contentious issue. It was suggested that combining the acuity levels diluted the specialist focus of the acute stroke unit, with continual negotiations required to maintain suitably qualified nurses to staff the stroke unit and protect existing nurse‐patient ratios:

*So it had its own FTE of staff who only worked in the stroke unit and that got degraded as everyone got mixed in. So, all the expertise within stroke got dispersed amongst the ward and it meant that you could have someone in the stroke unit who essentially had no experience working with acute stroke patients*. *[Variable Adherence 1]*

It was also perceived that the change in acuity resulted in a change in leadership. In the acute care setting, nurses were perceived to lead patient care. Whereas in the sub‐acute setting, it was perceived that allied health was directing patient care:

*It might take 45 min to get someone dressed, showered and ready so they can attend their 10 am physio session. But the nurse might then have their eye off the ball when someone is acutely deteriorating and is not able to respond in a timely manner. You lose that primary focus of the nurse leading the care to subacute focus where Allied Health direct the care*. *[High Adherence 2]*

Stroke Coordinators from hospitals with low adherence that did not have a stroke unit managed patients with stroke on a mixed general medicine ward or the patients could be dispersed throughout the hospital to any bed with telemetry capacity. The mixed ward included both acute and sub‐acute stroke patients. Although the Nurse Unit Manager made every effort to keep the acute and sub‐acute stroke patients at opposite ends of the shared ward, there was no delineation of staff or treatment phase‐specific protocols.

###### 4.2.2.1.3 Previous FeSS Intervention Study Participation

Two of the hospitals with high FeSS Protocol adherence and one of the hospitals with variable adherence had previously participated in a FeSS Intervention study. None of the hospitals with low FeSS Protocol adherence had participated in any of the FeSS Intervention studies. When Stroke Coordinators were asked to recall the implementation strategies used during the studies, their responses were non‐specific, or they could not remember:

*The designated clinical champions during that trial have continued, there was probably only myself and one of the other stroke nurses who is still here working. You sort of forget about it now because it's sort of just, you know, business as usual* *[High Adherence 1]*

*I remember a lot of the training that happened particularly in the emergency department, I think there was a bit of uptake, particularly when it was happening and then it just died off again, because there weren't champions on the floor pushing that stuff*. *[Variable Adherence 1]*

It was not clear if the limited clinician recall of the strategies used during these studies was indicative of the time that had passed since participating in the study and/or the successful embedding and normalisation of routine use of FeSS Protocols in their hospitals.

##### 4.2.2.2 Mapping to Factors From the Sustainability of Innovation Framework

Management support and recognition of the expertise required to care for patients with acute stroke are important *Workforce Factors* for sustainability. The existence of a designated space within the organisation for management of this critical acute stroke period acknowledges the specialist skills required of staff who work in this space. Similarly, having dedicated staff who have achieved the required competency in speciality skills to work in these environments is also a *workforce factor* at the individual level that relates to their personal motivation, capacity, and competence. Centralising the foci of professional knowledge facilitates assimilation of the innovation as it is more likely to be consistent with the values and needs of the staff working there (Greenhalgh et al. 2004). Examples presented by Stroke Coordinators of potential threats to sustainment of implementation strategies include inconsistent staffing or high staff turnover, decline in morale (e.g., relocation of stroke unit), and staff attitudes and perceptions (mismatch of co‐located clinical specialities) (Fox et al. 2015).

Previous participation in a FeSS Intervention study is relevant to *Political factor*s for sustainability. A focus on one particular policy or directive from the organisation at the early stage of implementation (such as agreement to participate in a clinical trial), can increase the chances of sustained implementation. To participate in one of the FeSS Intervention studies, agreement across the relevant departments would have been required for institutional and ethical approval before commencement. The additional time to negotiate these approvals prior to implementation, is likely to have positively influence sustained use of FeSS Protocols. The Stroke Coordinator from a hospital with low adherence also highlighted the importance of understanding other competing organisational directives before trying to implement the Protocols. The hospital did not have a specialist stroke unit or designated area for stroke patients. The Stroke Coordinator described some major resistance to implement any form of stroke pathway or protocol due to the competing national directive to immediately transfer patients out to a facility that did have a stroke unit:

*There's this big focus on making sure referrals were done, everyone would be spending all of their time ringing and faxing and you know my frustration then was but, in the meantime, let's just do some basic stuff … I was repeatedly told you can't have a stroke pathway because you don't have a stroke service* *[Low Adherence 2]*

This obviously becomes an issue if stroke‐unit beds at other hospitals are at capacity and the patient with stroke must remain on site in a non‐specialist area with no pathway to guide recommended care in this acute period.

## 5 Discussion

Despite the proven benefits of the evidence‐based FeSS Protocols supported by a strong recommendation in clinical practice guidelines, their consistent application remains suboptimal. Understanding factors that contribute to clinical variation in acute stroke care is critical to improving adherence and ultimately patient outcomes. There are challenges in implementing and sustaining evidence‐based care where different disciplines and departments are involved. Exploration of clinician perspectives of individual and organisational factors influencing sustained use of FeSS Protocols in Australian hospitals generated two themes constructed from six sub‐themes. These are mapped to four factors from the Sustainability of Innovation Framework. Theme 1: *Stroke Coordinator as sustainability champions and boundary spanners* [maintenance of implementation strategies; fostering working relationships, communication and influence] mapped to workforce, organisational, and innovation‐specific factors. Theme 2: *Hospital executive and middle management respect of stroke speciality* [designated area for stroke care; recognition of stroke specialist nursing skills; previous FeSS Intervention study participation] mapped to workforce and political factors. Stroke Coordinators from hospitals with consistently high adherence were from stroke units and were supported by other clinical champions. Hospitals with consistently low and variable adherence reported difficult interdepartmental working relationships and competing organisational directives.

The champion role is a powerful implementation strategy frequently used in research (Greenhalgh et al. 2004). The term is often used interchangeably with ‘opinion leader’ or ‘change agent’ despite the differences in these roles, contributing to a lack of clarity about their effectiveness. Research usually focuses on the role of champions in relation to implementation rather than how the role continues or changes longer term, or in relation to sustainability (Miech et al. 2018). The Stroke Coordinator's role in implementation was expected given the leadership status of this senior nursing position. However, their informal role in the sustainment of implementation strategies that included (but was not limited to): maintenance of hospital‐specific policies and protocol documents, education and training, coordination of data collection for Audits and other repositories, and the responsibility of delivering feedback of these audit results to multiple disciplines and departments was a key finding. The Stroke Coordinator's interpersonal skills and ability to foster working relationships with other disciplines and departments are critical to their dual role of boundary spanner, as cooperation from multiple stakeholders is required for sustained use of FeSS Protocols. Boundary spanning activities in an American study of advanced practice professionals (nurse practitioners and physician assistants) who act in Stroke Coordinator roles have also been credited for maintaining sustained engagement in acute stroke care (Rattray et al. 2017).

FeSS Protocol adaptations to suit local context are consistent with literature on sustainability of healthcare innovation that sustained use of the FeSS Protocols as initially implemented is not a static event, but a process that must be continually negotiated among members of an organisation long after initial implementation (Fox et al. 2015; Chambers et al. 2013). This type of continual improvement (adaptations) after initial implementation for optimisation in response to local context enables sustainability (Chambers et al. 2013). The Stroke Coordinator's leadership and collaboration skills can positively influence this process with effective communication channels to develop a shared understanding and values in relation to the use of FeSS Protocols. One example of these efforts is the feedback of audit results across different departments and disciplines to drive engagement for the use of FeSS Protocols.

There is evidence of other nurse leader champions' influence on the sustainability of best‐practice guidelines in hospitals for falls prevention, pressure ulcer prevention, and pain management (Fleiszer et al. 2016). Champions are also included as a factor in the Integrated Sustainability Framework (Shelton et al. 2018), and a recent systematic review of evidence‐based intervention sustainability strategies (Hailemariam et al. 2019). However, a more recent scoping review found the role and responsibilities of the champion were poorly defined (Ferren et al. 2022). Similarly, despite national and international recommendations for a dedicated Stroke Coordinator position as an integral part of a hospital's stroke service (Dusenbury et al. 2023; Stroke Foundation 2019), their role and scope of practice are often inconsistently defined (Purvis et al. 2021). This inconsistency is evident in one Stroke Coordinator's lack of awareness of participation in the audit in contrast to other Stroke Coordinators who led this quality improvement activity. Furthermore, it highlights that the audit results were not shared with nursing staff, which may hinder their engagement with evidence‐based care.

It is evident from this process evaluation that Stroke Coordinators cannot effect practice change alone. The use of a single staff member model, highlighted as a workforce factor for sustainability, may impact sustained use of FeSS Protocols. Having more than one Stroke Coordinator or designated stroke champions in other departments was identified as a contributing factor to the successful sustainment of implementation strategies for the use of FeSS Protocols. The effectiveness of multiple champions compared to single champions has been recognised in implementation research (Miech et al. 2018) and was also highlighted in the study of advanced practice professionals acting in Stroke Coordinator roles (Rattray et al. 2017).

There were numerous strategies identified for embedding FeSS Protocols into site‐specific clinical practices, policies and processes. However, where patients with stroke were not cared for in a designated area where care is centralised and managed by the multidisciplinary team, protocols were seen as a guideline rather than mandatory. Similarly, the inclusion of acute and sub‐acute stroke care in the same designated workspace was not viewed positively by interview participants, due to the lack of distinction in clinical areas. Stroke‐unit care remains the single most important recommendation in the national stroke guidelines (Stroke Foundation 2019). Overcoming this barrier requires management support and acknowledgement of the need for a distinct area with acute stroke care protocols, staffing ratios and the skillset required for staff to work in respective clinical areas.

Consistent with the literature on diffusion of innovations, the stroke units that were able to sustain consistently high FeSS Protocol adherence were stand‐alone units with decentralised decision‐making structures (Greenhalgh et al. 2004). The length of time a stroke unit had been established may account for some of the variation in FeSS Protocol adherence; however, investigating this was beyond the scope of this study. Regardless, international translation of FeSS Protocols in Europe (*n* = 64 hospitals, 17 countries) has demonstrated that the protocols can be successfully implemented by both stroke‐unit and non–stroke‐unit hospitals, noting that the establishment of dedicated stroke units is not always possible in smaller regional and remote hospitals (Ding et al. 2024). A designated area for stroke patients within a general or neurology ward has been demonstrated in other studies to maintain high standards of acute stroke care, particularly when using nursing ‘care bundles’ similar to the way the FeSS Protocols are packaged (three to five evidence‐based interventions) (Klinke et al. 2025). These examples highlight the instrumental role nurses can play in advancing stroke care through the use of nurse‐led protocols. Maintaining high‐quality local services for patients with stroke unable to access stroke‐unit care has been identified as a challenge in this study and in the literature. Efforts are ongoing to determine what elements of organised stroke care can be implemented to make the largest gain in these lower‐resourced settings (Langhorne et al. 2020).

### 5.1 Strengths and Limitations of the Work

Strengths of this study include the use of end‐user perspectives, given that clinician acceptance has been identified as a key factor for sustainability (Wade et al. 2014). Furthermore, the choice of Stroke Coordinators as the most likely personnel to be sufficiently knowledgeable regarding factors that may influence adherence to FeSS Protocols is supported by the finding of a median of 7.5 years of service in this leadership role. Those participants with fewer years of service were able to nominate a secondary person for interview who had either filled this role before them or been directly involved with the implementation of FeSS Protocols in their hospital. The secondary interviews enhanced the reliability and applicability of the data obtained during the primary interviews and improved the generalisability of data in relation to the hospital. Additional strengths consist of the purposive sampling design to efficiently analyse sustained adherence to FeSS Protocols and the extended time period over four audit cycles that spanned a 6‐year period to explore sustainability. Sampling by adherence levels enabled focused data collection from participants who were able to provide valuable insights into their consistent ranking of high or low adherence across the audit cycles. Additionally, the inclusion of hospitals with variable adherence to FeSS Protocols generated some understanding of why this inconsistency may have occurred. The substantial 6‐year period for evaluation of an evidence‐based intervention at recommended multiple time points after scale‐up is noteworthy, given the criticism of many studies attempts to evaluate sustainability at only 1–2 years post‐implementation (Braithwaite et al. 2020). Although we achieved representation from Stroke Coordinators across all Australian states and territories with the exception of one, the specialist nature of this clinical role and purposeful selection on other criteria may limit the representativeness of findings for other health professionals involved in stroke care.

Using a theoretical framework tailored to sustainability, and grounded in other established frameworks (Greenhalgh et al. 2004; Chambers et al. 2013), facilitated a comprehensive understanding of the factors for sustainability. Although the data only mapped to four of the five factors that make up this framework, this was not considered a limitation. *Financial* factors were not identified as Stroke Coordinators are generally not tasked with departmental budget management. Similarly, authors of another study that used this framework to evaluate sustainability of Nurse Practitioners in Emergency Departments, also did not report findings that were relevant to this factor (Fox et al. 2018).

### 5.2 Recommendations for Further Research

Future research on the national stroke‐unit accreditation, which commenced in 2022, will be of interest to evaluate any impact this process may have on adherence to clinical practice guidelines (Kleinig and Murphy 2024). Additionally, there is a clinical trial underway in Australia and New Zealand that involves a multi‐component implementation strategy using external remote facilitation to support healthcare teams in adopting FeSS Protocols (Fasugba et al. 2023). This approach may prove particularly beneficial for regional and remote areas and will direct future efforts in determining the amount of external support required to assist hospitals in implementing FeSS Protocols.

### 5.3 Implications for Policy and Practice

The Stroke Coordinator is recognised for their role in ensuring coordinated, best‐practice care (Stroke Foundation 2019), but their key role as sustainability champions has yet to be acknowledged in the literature. This finding is important not only for the sustained use of FeSS Protocols but also likely relevant to other stroke clinical practice guidelines. It is recommended that all primary and comprehensive stroke services in Australia have a dedicated Stroke Coordinator role. Despite this, according to the 2023 national audit of acute stroke services (*n* = 107 participating hospitals), only 70 of 86 (81%) services with stroke units and 11 of 21 (52%) services without a stroke unit reported having a dedicated Stroke Coordinator role (Stroke Foundation 2023). Highlighting the value of this nursing leadership position by acknowledging their critical role in sustainability strengthens the rationale for staffing of the Stroke Coordinator role in all stroke services.

## 6 Conclusion

This process evaluation after scale‐up strategies to maximise nationwide use of FeSS Protocols has identified several key factors for sustaining adherence to this clinical practice guideline. Importantly, it has recognised the pivotal role of the Stroke Coordinator in the sustainment of implementation strategies enabled by their boundary‐spanning skills. The unique skillset of these stroke leaders should be acknowledged by health services to guide future implementation strategies to optimise in‐hospital acute stroke care and improve patient outcomes.

## Author Contributions

All authors have agreed on the final version and meet at least one of the following criteria (recommended by the ICMJE): (1) substantial contributions to conception and design, acquisition of data, or analysis and interpretation of data; (2) drafting the article or revising it critically for important intellectual content.

## Conflicts of Interest

Corresponding author and co‐authors S.M., D.A.C., S.D., K.C., E.M. and O.F. are investigators for the QASC Research Program (QASC, QASCIP, T^3^ and QASC Europe Studies). K.H. reports a relationship with Stroke Foundation Australia that includes employment. Co‐author D.A.C. is responsible for the independent and alloy analysis of National Stroke Audit data on behalf of the Stroke Foundation.

## Supporting information


**Data S1:** jan70125‐sup‐0001‐DataS1.pdf.

**Data S2:** jan70125‐sup‐0002‐DataS2.docx.

## Data Availability

The data that support the findings of this study are available on request from the corresponding author. The data are not publicly available due to privacy or ethical restrictions.
