# Supplementary material for: Stroke Coordinators' Perspectives on Sustaining Use of Fever, Sugar (Hyperglycaemia) and Swallow (FeSS) Protocols. Process Evaluation Using a Sustainability Framework
Source: J Adv Nurs. 2025 Aug 5;82(4):3522–36. doi: 10.1111/jan.70125 (PMC12994674; doi:10.1111/jan.70125)
Supplement: Supplementary file 2 — Data S2: jan70125‐sup‐0002‐DataS2.docx. [file JAN-82-3522-s001.docx]

| Questions mapped to *components* **Greenhalgh et al, (2004)** Conceptual Model for Diffusions of Innovations in Health Service Delivery and Organisations^1^ |
| --- |
| Questions mapped to *factors* **Fox et al,(2015)** Sustainability of Innovation framework^2^ |

**Interview Guide:** Stroke Coordinator /Stroke Clinicians

**Aim:**

To identify Stroke Coordinator /Stroke Clinicians views on the factors that may have influenced the introduction, implementation and embedding of clinical protocols for the management of Fever, Sugar (hyperglycaemia) and Swallow (**F**e**SS**) complications after stroke in their stroke service.

**Preamble**

Thank you for agreeing to speak with me today. My name is XXXX and I am one of the researchers conducting interviews with Stroke Clinicians directly involved in acute stroke care and who are knowledgeable about the use of **F**e**SS** Protocols in their hospital.

****Choose relevant Option A or B***

**Option A:** You have been selected for this interview because of your role as Stroke Coordinator at (*insert name of hospital*). As such, we anticipate that you will be able to give an informed perspective about your role and on factors that you think may have influenced the introduction of and uptake and sustainability of the **F**e**SS** Protocols.

**Option B:** You have been identified through our interview with your hospital’s Stroke Coordinator as a stroke clinician (XXXX insert discipline and/or role if known) at (*insert name of hospital*) who may be able to give an informed perspective about factors that you think may have influenced the introduction of and uptake and sustainability of clinical protocols for the management of Fever, Sugar (hyperglycaemia) and Swallow (**F**e**SS**) complications after stroke in your stroke service. This includes whether they were actually implemented at your hospital, or only partially implemented, and/or implemented but then discontinued.

**Recording instructions**

If you are agreeable, I will take notes and record the interview so that I don’t miss any details and can carry on an attentive conversation with you. All interviews are confidential, and recordings and transcripts of the interview will be anonymised and you and your hospital will not be identified in any publication.

Do you have any questions?

**Consent form instructions**

Check that the interviewee has read the participant information sheet and read and signed consent forms. They keep a copy and the researcher keeps a copy as well.

**PART A**

**1. Role of interviewee**

1.1 Clinical designation of interviewee at time of interview_______________________________________

1.2 Length of service within organisation __________________________

1.3 Years clinical experience and brief background (how long have they worked in stroke specialty)

1.4 Did your hospital implement clinical protocols for the management of Fever, Sugar (hyperglycaemia) and Swallow (**F**e**SS**) complications after stroke? _________________________

*If No (or don’t know) got to **PART C**

1.5 Have you been involved in any way in implementing **F**e**SS** Protocols at any point? Y/N?

*If Yes what was your role exactly (e.g. education, distributing materials, obtaining resources, utilising them in clinical practice etc)

1.6. Can you recall the time period that you were involved in implementation?

(*Need the year at least to be able to document how long after implementation the interview is taking place)

**PART B**

**Implementation (*sites not enrolled in FeSS Intervention study)**

2.1 What efforts have been made to introduce the **F**e**SS** Protocols in your stroke service?

(e.g. in-service education, added to stroke pathway, signs up in work space). If yes to education sessions- are these revisited often during in-service calendar year? Part of new staff training?

| Implementation and routinisation (human resource issues, team based training, funding) |
| --- |
| Political factors (staff involvement in implementation and decision making) |

2.2. Were you able to try out different aspects of the FeSS Protocols before or during their introduction (i.e. was it broken down into stages or all at once)?

| The Innovation (Trialability, Reinvention, complexity) |
| --- |
| Organisational factors (Refining of innovation) |

2.3 Are there any modified versions of any of the Fever, Sugar, Swallow Protocols in use? (e.g. different insulin regime, only monitor for day of admission not full 72hrs)-

| The Innovation (Reinvention, risk) |
| --- |
| Organisational factors (Refining of innovation) |

2.4 Was implementation of the **F**e**SS** Protocols led by a specific health discipline?

(e.g. nursing/medical/speech)

| Adoption by individuals (Context-specific psychological antecedents, the adoption decision)  Diffusion and dissemination (Champions) |
| --- |
| Workforce factors (single staff member model, staff attrition, inadequate staffing)  Financial factors (funding)  Political factors (staff involvement in implementation and decision making) |

2.5 What were the feelings of the stroke service team in general about the introduction/training in the use of the protocols? Was everyone included in the training? Was/is there enough training? What methods were used? (*Probe: Was/is there any concerns from stroke team towards any aspect of adopting the FeSS Protocols? What are these concerns? Internal, external factors)

| System readiness for innovation (Power balances [support and advocacy; peer opinion leader (Communication and influence)  Assimilation by the system (the assimilation)  Diffusion and dissemination (Network structure, Opinion leaders)  Implementation and routinisation (intraorganizational communication, human resource issues, team-based training, funding) |
| --- |
| Workforce factors (communication and networking strategies) |

- - 1. Were management supportive of the FeSS Protocols and implementation process?

(e.g. supervisors/ heads of department for nursing/medical/allied health/speech pathology).

(Probe for any suggestion of a ‘hands on approach’?e.g. vocal support for their use at MDT meetings, attendance at training)

| System Antecedents for innovation (Structural determinants of innovativeness [slack resources]; Receptive context to change [leadership and vision/ good managerial relations]) System readiness for innovation (Dedicated time and resources)  Diffusion and dissemination (Opinion leaders)  Implementation and routinisation (leadership and management) |
| --- |
| Financial factors (funding)  Workforce factors (inadequate staffing)  Political factors (upper management and organisational support) |

2.7 Are use of the **F**e**SS** Protocols formalised (e.g. part of the EMR or stroke care pathway, policy) or informal (e.g. everyone just knows that you have to monitor for fever, sugar, swallow complications but it’s not documented as part of their predicted care plan)?

(*Probe for details for each variable specifically (fever, sugar, swallow. Are there designated roles when implementing the **F**e**SS** Protocols (e.g. is everyone competent in performing swallow screens, are they completely nurse led)?

| The Innovation (compatibility)  System antecedents for innovation (Absorptive capacity for new knowledge “learning organisation culture”)  Assimilation by the system  Implementation and routinisation (leadership and management) |
| --- |
| Political factors (policy) |

- 1. What do you think have been the barriers and/or enablers to the introduction of the FeSS Protocols in your stroke service? (*Probe for resolved and intractable and each protocol specifically [fever, sugar, swallow])

***Barriers:***

***Enablers:***

- 1. What do you think, if anything, could have helped to resolve the intractable barriers/promote the enablers ? (*Probes/prompts barriers raised in FeSS Intervention studies e.g. medical team buy in, no medical champion? Beliefs in evidence, outside scope of practice, resources [ training, workload, equipment, time, budget]

***Barriers:***

***Enablers:***

3.0 Do you think the **F**e**SS** Protocols are simple and easy to implement?

(*Prompt: Do you think the FeSS Protocols are easy to teach or show new staff? When seeking advice about use of the FeSS Protocols who would you normally direct these queries to? What processes do they have to train staff new to the ward about FeSS? (e,g orientation module, on the job training, competency to be completed with educators?)

| The Innovation (compatibility,complexity, task issues, knowledge to use it) |
| --- |
| Innovation-specific factors (acceptability, quality) |

**Implementation process**

3.1 When considering use of the **F**e**SS** Protocols in clinical practice do you think communication regarding their use amongst the multi-disciplinary team was/is effective?

(*Prompts: Stroke team responsive to treatment of fever, sugar, swallow complications, topic for discussion in team meetings, communication to new staff and reminders to existing staff)

| System Antecedents for Innovation (absorptive capacity for new knowledge [enablement of knowledge sharing via internal networks])  Implementation and routinisation (intraorganizational communication) |
| --- |
| Organisational factors (communication, meetings teamwork) |

3.2 Is there a process for any kind of feedback in relation to the use of the **F**e**SS** Protocols?

(*Prompts: internal performance audits [AuSCR optional dataset for **F**e**SS**], Stroke Foundation audit results, feedback from speech department, Endocrinology referrals, are they referred to in MDT meetings)?

(*Probe: What are the expectations within your organisation in regard to use of the FeSS protocols? Are there reprimands if the protocol is not followed/acknowledgement when they are used correctly? From whom)?

| System Antecedents for Innovation (Receptive context to change [high quality data capture])  System readiness for innovation (Monitoring feedback, support and advocacy, capacity to evaluate the innovation)  Adoption by individuals (concerns in established users)  Implementation and routinisation (feedback) |
| --- |
| Innovation-specific factors (evaluation measures)  Workforce factors (quality monitoring and feedback) |

**Innovation**

4.1 What do you think or see are the benefits of the **F**e**SS** Protocols? (*PROBE: Have they had any impact on referral volume etc, Do you think the evidence is clear about the value of them for improving patient care and outcomes?)

| The Innovation (relative advantage) |
| --- |
| Innovation-specific factors (acceptability, quality) |

4.2 Are there any risks to the patient or staff that you can think of in relation to the FeSS Protocols? If so do these outweigh the benefits for their use? (e.g. hypoglycaemia, acting outside scope of practice)

| The Innovation (risk)  System Readiness for Innovation (innovation-system fit, assessment of implications |
| --- |
| Innovation-specific factors (safety) |

4.3 How do the **F**e**SS** Protocols fit within your hospital’s normal ward routine (e.g. vital sign scheduling)? (*Probe: If their use has discontinued is it due to this or would they require significant ward level changes to accommodate use of the protocols; if so, what kind of changes?).

| System readiness for Innovation (Innovation-system fit)  The Innovation (compatibility)  Implementation and routinisation |
| --- |
| Workforce factors (processes in place to support and evaluate) |

4.4 When considering your stroke service, is there an emphasis on evidence-based care (e.g. getting clinical guidelines into clinical practice, removing outdated practices).

| System antecedents for innovation (Absorptive capacity for new knowledge “learning organisation culture”) |
| --- |
| Innovation specific factors (support/barriers to the innovation)  Political factors (staff involvement in implementation and decision making) |

4.5 Do you think there is an awareness of the strong recommendation for use of **F**e**SS** Protocols in the National Clinical Guidelines for Stroke Management?

| Diffusion and dissemination (Boundary spanner) |
| --- |
| Political factors (staff involvement in implementation and decision making) |

4.6 What about nurse-led projects in your stroke service? Are they common? Can you provide some examples?

| System antecedents for innovation (structural determinants of innovativeness [slack resources, absorptive capacity for new knowledge]) |
| --- |
| Political factors (staff involvement in implementation and decision making) |

**Communication and influence**

5.1 Are you aware of other stroke services experience in implementing **F**e**SS** Protocols? Are they a topic likely to be discussed at professional group meetings? (e.g. ASNEN, Stroke Society Australasia)

| Diffusion and dissemination (Homophily, Opinion leaders, champions, boundary spanners)  The Outer Context: Interorganisational networks and collaboration (Informal interorganisational networks) |
| --- |
| Organisation factors (networking external organisations) |

5.2 Were there any changes in the organisation at any point that may have had a positive or negative impact upon the implementation of the protocols? (*Probe for sustainability)

| Assimilation by the system |
| --- |
| Workforce factors (staff role changes)  Organisational factors (alignment, staff involvement)  Innovation-specific factors (fluidity, adaptability) |

**Final thoughts and comments**

Is there anything else that you would like to add? Recommendations to others that are planning to implement the FeSS Protocols in their hospitals?

**Thank you for your valuable time and input**

**PART B**

**Implementation (*sites enrolled in FeSS Intervention study)**

2.1 Do you recall what strategies were used during the XXXX study to introduce and implement the FeSS protocols? (e.g. was there a clinical champion, meetings with opinion leaders, education sessions)

| Linkage among components of the model (Role of the change agency) |
| --- |
| Political factors (staff involvement in implementation and decision making) |

2.2 Did these same strategies continue after trial completion. (e.g. clinical champion, education for new staff, spot auditing).

| Linkage among components of the model (Role of the change agency) |
| --- |
| Political factors (staff involvement in implementation and decision making) |

2.3 Have there been additional efforts or strategies to embed the **F**e**SS** protocols in your stroke service (either during or after the FeSS intervention study?) [ e.g. education sessions- are these revisited often during in-service calendar year? Part of new staff training, added to stroke pathway, signs up in work space, does FeSS belong to nurse leaders portfolio?] What processes do they have to train staff new to the ward about FeSS? (e,g orientation module, on the job training, competency to be completed with educators?)

| System antecedents for innovation (Absorptive capacity for new knowledge “learning organisation culture”) |
| --- |
| Political factors (staff involvement in implementation and decision making) |

2.4. Were you able to try out different aspects of the FeSS protocols before or during their introduction (i.e. was it broken down into stages or all at once)?

2.5 Are there any modified versions of any of the Fever, Sugar, Swallow protocols in use? (e.g. different insulin regime, only monitor for day of admission not full 72hrs- maybe better suited to your workplace?)

2.6 Was implementation of the **F**e**SS** protocols led by a specific health discipline

(e.g. nursing/medical/speech)?

2.7 What were the feelings of the stroke service team in general about the introduction/training in the use of the protocols? Was everyone included in the training? Was/is there enough training? What methods were used? (*Probe: Was/is there any concerns from stroke team towards any aspect of adopting the FeSS protocols? What are these concerns? Internal, external factors)

- 1. Were management supportive of the FeSS protocols and implementation process (e.g. supervisors/ heads of department for nursing/medical/allied health/speech pathology).

(Probe for any suggestion of a ‘hands on approach’?e.g. vocal support for their use at MDT meetings, attendance at training)

2.9 Are use of the **F**e**SS** protocols formalised (e.g. part of the EMR or stroke care pathway, policy) or informal (e.g. everyone just knows that you have to monitor for fever, sugar, swallow complications but it’s not documented as part of their predicted care plan)? (*Probe for details for each variable specifically (fever, sugar, swallow. Are there designated roles when implementing the **F**e**SS** protocols (e.g. is everyone competent in performing swallow screens, are they completely nurse led)?

1. What do you think have been the barriers and/or enablers to the introduction of the FeSS protocols in your stroke service? (Probe for resolved and intractable and each protocol specifically [fever, sugar, swallow])

***Barriers:***

***Enablers:***

- 1. What do you think, if anything, could have helped to resolve these intractable barriers/promote the enablers ? (*Probes/prompts barriers raised in FeSS Intervention studies e.g. medical team buy in, no medical champion? Beliefs in evidence, outside scope of practice, resources [ training, workload, equipment, time, budget]

***Barriers:***

***Enablers:***

3.2 Do you think the **F**e**SS** protocols are simple and easy to implement?

(*Prompt: Do you think the FeSS protocols are easy to teach or show new staff? When seeking advice about use of the FeSS protocols who would you normally direct these queries to? What processes do they have to train staff new to the ward about FeSS? (e,g orientation module, on the job training, competency to be completed with educators?)

(*Prompt: Do you think the FeSS Protocols are easy to teach or show new staff? When seeking advice about use of the FeSS Protocols who would you normally direct these queries to?

**Implementation process**

4.1 When considering use of the **F**e**SS** protocols in clinical practice do you think communication regarding their use amongst the multi-disciplinary team was/is effective? (*Prompts: Stroke team responsive to treatment of fever, sugar, swallow complications, topic for discussion in team meetings, communication to new staff and reminders to existing staff)

4.2 Is there a process for any kind of feedback in relation to the use of the **F**e**SS** protocols?

(*Prompts: internal performance audits [AuSCR optional dataset for **F**e**SS**], Stroke Foundation audit results, feedback from speech department, Endocrinology referrals, are they referred to in MDT meetings)?

(*Probe: What are the expectations within your organisation in regards to use of the FeSS protocols? Audit results? Are there reprimands if the protocol is not followed/Acknowledgement when they are used correctly? From whom)?

**Innovation**

5.1 What do you think or see are the benefits of the **F**e**SS** protocols?

(*PROBE: Have they had any impact on referral volume etc, Do you think the evidence is clear about the value of them for improving patient care and outcomes?)

5.2 Are there any risks to the patient or staff that you can think of in relation to the FeSS protocols? If so do these outweigh the benefits for their use? (e.g. hypoglycaemia, acting outside scope of practice)

5.3 How do the **F**e**SS** protocols fit within your hospital’s normal ward routine (e.g. vital sign scheduling)? (*Probe: If their use has discontinued is it due to this or would they/did they require significant ward level changes to accommodate use of the protocols)

5.4 When considering your stroke service, is there an emphasis on evidence-based care (e.g. getting clinical guidelines into clinical practice, removing outdated practices).

5.5 Do you think there is an awareness of the strong recommendation for use of **F**e**SS** protocols in the National Clinical Guidelines for Stroke Management? )

5.6 What about nurse-led projects in your stroke service? Are they common? Can you provide some examples?

**Communication and influence**

6.1 Are you aware of other stroke services experience in implementing **F**e**SS** protocols? Are they a topic likely to be discussed at professional group meetings? (e.g. ASNEN, Stroke Society of Australasia)

6.2 Were there any changes in the organisation at any point that may have had a positive or negative impact upon the implementation of the protocols? (*Probe for sustainability)

**Final thoughts and comments**

Is there anything else that you would like to add? Recommendations to others that are planning to implement the FeSS protocols in their hospitals?

**Thank you for your valuable time and input**

**PART C**

**For hospitals that answered that there had been* ***NO attempts*** *or they* ***don’t know*** *of any attempts to implement clinical protocols for the management of Fever, Sugar (hyperglycaemia) and Swallow (****F****e****SS****) complications after stroke*

- 1. What do you think have been the barriers to the introduction of **F**e**SS** protocols in your stroke service? (*Probe for resolved and intractable and each protocol specifically [fever, sugar, swallow])
  2. What do you think, if anything, could have helped to resolve these intractable barriers/promote the enablers? (*Probes/prompts barriers raised in FeSS Intervention studies e.g. medical team buy in, no medical champion? Beliefs in evidence, outside scope of practice, resources [ training, workload, equipment, time, budget]
  3. Are you aware if the idea of introducing the FeSS protocols has ever been revisited?

2.4 Have you been able to try out any aspect of the FeSS protocols (e.g. only the fever or only the swallow screening)?

2.5 Are there any modified versions of any of the Fever, Sugar, Swallow protocols in use? (e.g. different insulin regime, only monitor for day of admission not full 72hrs).

- 1. Are you aware of any input from management regarding the use of protocols for the management of fever, sugar or swallow complications after stroke?

| System Antecedents for innovation (Structural determinants of innovativeness [slack resources]; Receptive context to change [leadership and vision/ good managerial relations]) System readiness for innovation (Dedicated time and resources)  Diffusion and dissemination (Opinion leaders)  Implementation and routinisation (leadership and management) |
| --- |
| Financial factors (funding)  Workforce factors (inadequate staffing)  Political factors (upper management and organisational support) |

2.7 What do you think or see are the benefits of the FeSS protocols? (*PROBE: Do you think the evidence is clear about the value of them for improving patient care and outcomes?)

2.8 Are there any risks to the patient or staff that you can think of in relation to the FeSS protocols? If so do these outweigh the benefits for their use? (e.g. hypoglycaemia, acting outside scope of practice)

2.9 If you are aware of the FeSS protocols, do you think they are simple and would be easy to implement? Who would you direct any queries to regarding their use? (e.g. another health service? professional network)

3.0 Do you receive any feedback regarding your hospitals performance in the national audit reports? If so, do you recall any feedback in relation to FeSS and how your service performs in this area generally? (*Prompts :Does your service conduct internal performance audits or contribute to a stroke registry?)

| System Antecedents for Innovation (Receptive context to change [high quality data capture])  System readiness for innovation (Monitoring feedback, support and advocacy, capacity to evaluate the innovation)  Adoption by individuals (concerns in established users)  Implementation and routinisation (feedback) |
| --- |
| Innovation-specific factors (evaluation measures)  Workforce factors (quality monitoring and feedback) |

3.1 How would the FeSS protocols fit within your hospital’s normal ward routine (e.g. vital sign scheduling)? (*Probe: would they require significant ward level changes to accommodate use of the protocols)

3.2 When considering your stroke service, is there an emphasis on evidence-based care (e.g. getting clinical guidelines into clinical practice, removing outdated practices).

3.3 Do you think there is an awareness of the strong recommendation for use of FeSS protocols in the National Clinical Guidelines for Stroke Management? )

3.4 What about nurse-led projects in your stroke service? Are they common? Can you provide some examples?

**Communication and influence**

4.1 Are you aware of other stroke services experience in implementing FeSS protocols? Are they something likely to be discussed at professional group meetings? (e.g. ASNEN, Stroke Society)

4.2 Were there any changes in the organisation at any point that may have had a positive or negative impact upon the implementation of FeSS protocols? (*Probe for sustainability)

Final thoughts and comments

Is there anything else that you would like to add? Recommendations to others that are planning to implement the FeSS protocols in their hospitals?

**Thank you for your valuable time and input**

1. Greenhalgh T, Robert G, Macfarlane F, Bate P, Kyriakidou O. Diffusion of Innovations in Service Organizations: Systematic Review and Recommendations. *The Milbank Quarterly* 2004; **82**(4): 581-629.

2. Fox A, Gardner G, Osborne S. A theoretical framework to support research of health service innovation. *Australian Health Review* 2015; **39**(1): 70.
